# Interaction between 17β-estradiol, prolactin and human papillomavirus induce E6/E7 transcript and modulate the expression and localization of hormonal receptors

**DOI:** 10.1186/s12935-019-0935-6

**Published:** 2019-09-02

**Authors:** Inocencia Guadalupe Ramírez-López, Adrián Ramírez de Arellano, Luis Felipe Jave-Suárez, Christian David Hernández-Silva, Mariel García-Chagollan, Jorge Hernández-Bello, Edgar I. Lopez-Pulido, José Macias-Barragan, Margarita Montoya-Buelna, José Francisco Muñoz-Valle, Ana Laura Pereira-Suárez

**Affiliations:** 1 Centro Universitario de Ciencias de la Salud, Universidad de Guadalajara, Guadalajara, Jalisco Mexico; 2 Laboratorio de Inmunología, Departamento de Fisiología, Centro Universitario de Ciencias de la Salud, Universidad de Guadalajara, Sierra Mojada # 950, Colonia Independencia, CP 44340 Guadalajara, Jalisco Mexico; 3 Instituto de Investigación en Ciencias Biomédicas, Centro Universitario de Ciencias de La Salud, Universidad de Guadalajara, Guadalajara, Jalisco Mexico; 4 División de Inmunología, Centro de Investigación Biomédica de Occidente (CIBO), Instituto Mexicano del Seguro Social (IMSS), Sierra Mojada 800, Col. Independencia, 44340 Guadalajara, JAL Mexico; 5 Departamento de Clínicas, Centro Universitario de Los Altos, Tepatitlán de Morelos, Universidad de Guadalajara, Guadalajara, Jalisco Mexico; 6 Departamento de Ciencias de La Salud CUValles, Universidad de Guadalajara, Guadalajara- Ameca Rd Km. 45.5, Ameca, Jalisco Mexico

**Keywords:** 17β-estradiol, Prolactin, HPV, E6/E7 oncogenes, ERα, ERβ, GPER, PRLR

## Abstract

**Background:**

Cervical cancer (CC) is the second most common cancer in less developed countries and the second leading cause of death by cancer in women worldwide. The 99% of CC patients are infected with the Human Papilloma Virus (HPV), being HPV16 and HPV18 infection the most frequent. Even though HPV is considered to be a necessary factor for the development of CC, it is not enough, as it requires the participation of other factors such as the hormonal ones. Several studies have demonstrated the requirement of estrogen and its receptors (ERα, ERβ, and GPER) in the precursor lesions progress towards CC. Also, prolactin (PRL) and its receptor (PRLR) have been associated with CC. The molecular mechanisms underlying the cooperation of these hormones with the viral oncoproteins are not well elucidated. For this reason, this study focused on analyzing the contribution of 17β-estradiol (E2), PRL, and HPV on the expression and localization of hormone receptors, as well as to evaluate whether these hormones may promote greater expression of HPV oncogenes and contribute to tumor progression.

**Methods:**

qPCR was used to evaluate the effect of E2 and PRL on the expression of E6 and E7 oncoproteins in HeLa and SiHa cervical cancer cells lines. HaCaT cells were transduced with the viral oncogenes E6 and E7 from HPV 16 and 18. ERα, ERβ, GPER, and PRLR expression and localization were evaluated by qPCR, Western blot and immunofluorescence.

**Results:**

E2 and PRL induce E6/E7 oncogenes expression in HeLa and SiHa cells. E6 and E7 oncogenes of HPV16/18 significantly increased the protein expression of ERα, GPER, and PRLR. ERβ was positively regulated only by E6 oncogenes of HPV16/18. Besides, some of these oncogenes modify the location of PRLR toward cytoplasm, and ERα, ERβ, and GPER mainly to the nucleus.

**Conclusion:**

Our studies suggest that the mutual regulation between E2, PRL, and HPV oncogenes could cooperate with the carcinogenesis process in CC.

## Background

### Introduction

CC is the second most common female malignancy worldwide and HPV is considered its main causal agent due to it is present in more than 99% of women with this disease. Nowadays, more than 200 genotypes of HPV are known; however, only 12 have been classified as high risk and they have been associated with CC. High-risk HPV-16 and -18 are present in over 85% of women with CC, this is why most of the molecular research is focused on these genotypes [1, 2].

HPV is a double-stranded DNA virus whose genome encodes 8 proteins, including the oncogenic E6 and E7 proteins, which have the ability to revert p53 and pRB suppressing functions [3].

Viral oncoproteins have an important role in the regulation of signaling cascades that initiate responses that determine cell survival and promote the development of CC. Recent studies have revealed that E6 and E7 oncoprotein facilitated the β-catenin nuclear accumulation, leading the activation of Wnt/β-catenin signaling cascade and acceleration of cervical carcinogenesis by activating genes that induce an increase in cell proliferation [4, 5]. E7 oncogene also induces the nuclear translocation of Smad proteins in a ligand-independent manner and interacted with MH1 Domain of SMAD3 to repress its transcription through TGF-β [6, 7].

Moreover, the E7 oncogene is related whit loss of TGF-β responsiveness via downregulation of TGF receptors in transgenic mice expressing this oncogene of HPV16 [8]. These findings suggest a prominent role of this HVP oncogenic proteins in the modulation of some signaling paths and receptors associated with cell proliferation [4].

Despite HPV is necessary for CC development, it is known that infection itself is not enough to establish a transition to cancer, meaning that other factors are important to collaborate, and induce cell malignant transformation [9].

17β-estradiol (E2) is a hormone that has been widely related to CC development. It has been proved in murine models expressing E6/E7 of HPV-16, that cancer is only attained when exposed chronically to this hormone [10]. Estrogen actions are exerted through the classical estrogen receptor α and β (ERα and ERβ), as well as the G protein-coupled estrogen receptor (GPER/GPR30) [11]. It has been described that ERα is involved with the CC development, due to when it is absent, cancer is not observed [12]. On the other hand, immunohistochemistry assays in human tissues have shown that ERα and ERβ increase its expression with the disease advance, which confirms the importance of this hormone in the evolution of the pathology [13]. On the other hand, GPER is highly expressed in tissues and CC cell lines and its activation is associated with inhibition of cellular proliferation [14, 15]. Moreover, a study showed that cytoplasmic GPER expression was correlated with a favorable prognosis in CC [16].

Prolactin (PRL) and its receptor (PRLR) are also important in CC; previous reports of our group of research have shown that PRLR expression in cervical tissue is increased as the disease advances towards cancer. Besides, the long isoform of PRLR was only expressed in CC, which suggests a differential signaling of this hormone-receptor axis between pre-malignant and cancer stages [13, 17]. Also, CC cell lines synthesize a 60 kDa PRL isoform, which decreases the apoptosis of these cells [18, 19].

E2 and PRL are hormones related to each other and can reciprocally regulate their expression and function. PRL induces genic expression of ERα y ERβ as well as activates ERα in absence of ligand [20, 21]; on the other hand, E2 can also modulate the expression of PRL due to its distal promoter has an estrogen receptor element (ERE) which can bind to ERα and ERβ [22], suggesting these hormones have a positive regulation loop.

It has been shown that E2 can regulate the expression of the E6 and E7 oncogenes of SiHa cells due to HPV16 contains sequences similar to ERE in the promoter region and the nuclear estrogen receptor can bind to these ERE [23].

It is unknown whether PRL may regulate the expression of oncogenes or, in turn, oncogenes may induce the expression of the receptors of E2 and PRL. Thus, this work is focused on evaluating the mutual regulation between hormones and oncogenes.

## Materials and methods

### Materials and reagents

The following reagents were employed for the transduction of E6 and E7 oncogenes in HaCaT cells: PCR system kit (cat. no. 11 732 650 001; Roche Applied Science), pGEM‑T easy cloning vector (cat. no. A1360; Promega Corporation, Madison, WI, USA), pLVX‑Puro lentiviral expression vector (cat. no. 632164; Clontech Laboratories, Inc., Mountain View,CA, USA), Lenti‑X 293T cells (cat. no. 632180; Clontech Laboratories, Inc.), Lenti‑X Lentiviral Expression system (Clontech Laboratories,Inc.), Lenti‑X GoStix (Clontech Laboratories, Inc.), RNeasy Plus Mini kit (Cat. 74136; QIAGEN Mexico, S. de R.L. de C.V., San Ángel, Mexico). For RT-qPCR we employed TRIzol reagent (Invitrogen, Carlsbad, CA), Transcriptor First Strand cDNA Synthesis kit (Roche Applied Science), LightCycler® FastStart DNA Master PLUS SYBR Green I kit (Roche Applied Science) and the LightCycler 2.0 instrument (Roche Applied Science). The cell culture medium employed was the Dulbecco's modified Eagle's medium (DMEM) high glucose supplement with GlutaMAX™, 10% of fetal bovine serum (FBS) or 10% of charcoal stripped FBS, penicillin G and streptomycin, all obtained from Gibco; Thermo Fisher Scientific, Inc. (Waltham, MA, USA); the stimuli were done with 17β-estradiol E8875, and prolactin human recombinant L4021 both from Sigma‑Aldrich; Merck KGaA (Darmstadt, Germany). Finally, the antibodies PRL-R sc-20992 rabbit polyclonal IgG, ER-α sc-542 rabbit polyclonal IgG, ERβ sc-373853 mouse monoclonal IgG2b, β-Actin sc-47778 mouse monoclonal, goat anti-rabbit IgG-HRP sc-2004, goat anti-mouse IgG-HRP sc-2005 1:10,000 all from Santa Cruz Biotechnology and Anti-G-protein (ab39742) coupled receptor 30 antibody from Abcam were employed for western blot and immunofluorescence.

### Cell culture conditions

Cervical cancer cell lines (HeLa and SiHa), were obtained from the American Type Culture Collection (Manassas, VA, USA); and non-tumorigenic human keratinocytes (HaCaT) cell line was kindly provided by Dr. Petra Boukamp from the German Cancer Research Center (DKFZ, Heidelberg, Germany). Cells were cultured in a water-jacketed incubator at 37 °C under an atmosphere of 95% air and 5% CO_2_ in culture medium and grown to 80% confluence. The growth culture medium consisted in DMEM medium supplemented with 10% FBS, penicillin (100 U/ml), streptomycin (100 μg/ml); when experiments involving hormonal stimuli were performed, supplemented media were prepared with FBS charcoal-stripped (10%).

### HaCaT cells transduced with E6 or E7 from HPV-16 and -18

The HaCaT cell lines transduced with E6 and E7 oncogenes and pLVX control were provided for the Drs. Luis Felipe Jave Suárez and Cristina Artaza Irigaray from Centro de Investigación Biomédica de Occidente (CIBO).

E6 and E7 cloning were performed by endpoint PCR for amplifying E6 and E7 open reading frame (ORF) from genomic DNA samples obtained from biopsies of patients infected with HPV-16 or -18. The following primer sets were employed: HPV 16 E6, forward: 5′ CAG ACA TTT TAT GCA CCA AA 3′, and reverse: 5′ CTC CAT GCA TGA TTA CAG C 3′; HPV 16 E7, forward: 5′ TAG AGA AAC CCA GCT GTA ATC A 3′, and reverse: 5′ AGG ATC AGC CAT GGT AGA TTA T 3′; HPV 18 E6, forward: 5′ AAT ACT ATG GCG CGC TTT GA 3′, and reverse: 5′ TTG CCT TAG GTC CAT GCA TAC T; and HPV 18 E7, forward: 5′ CGC AGA GAA ACA CAA GTA TAA T 3′ and reverse: 5′ GAT CAG CCA TTG TTG CTT A 3′. The four amplified products were independently cloned into a pGEM‑T Easy cloning vector and were employed for the transformation of TOP10 Chemically Competent E. coli by the technique of heat shock, the transformed bacteria were selected and then a restriction analysis with EcoRI was carried out and the genomes of the oncoproteins were sequenced and corroborated with the reference sequences reported in GenBank (HPV 16, accession no. K02718; HPV 18, accession no. AY262282; https://www.ncbi.nlm.nih.gov/genbank/); only HPV 16 E6 exhibited a substitution described in the AF402678 HPV 16 sequence (268T > G). Finally, the ORFs of the oncogenes were subcloned into a pLVX‑Puro lentiviral expression vector.

As for HaCaT cells infection, Lenti‑X 293T cells were employed to produce infectious viral particles through the Lenti‑X Lentiviral Expression system. These cells were independently transfected with pLVX‑Puro empty vector, pLVX‑HPV16E6, pLVX‑HPV16E7, pLVX‑HPV18E6 or pLVX‑HPV18E7 vectors, using the Lenti‑X HTX Packaging system. After 48 h post‑transfection, the supernatants with infectious viral particles were collected, the presence of the virus particles was determined using Lenti‑X GoStix. HaCaT cells were individually infected with 100 μl of each viral supernatant and then the transduced cells were selected with 1 μg/ml puromycin. The E6 and E7 expression levels in HaCaT cells were determined with reverse transcription‑quantitative PCR (RT‑qPCR) using the same primers used for cloning. For the RNA extraction, the RNeasy Plus Mini kit was used according to the manufacturer's protocol and then followed the procedure described in RT-qPCR.

### Hormone stimuli

The cells were grown into 6-well plates with DMEM medium supplemented with 10% charcoal stripped fetal bovine serum at approximately 80% confluence, after the hormones were added (17β-estradiol, 10 nM; and prolactin, 200 ng/ml) and stored in a water‑jacketed incubator at 37˚C in an atmosphere containing 5% CO_2_ for 4 h.

### Total RNA isolation and RT-qPCR

Total RNA was isolated using the phenol–chloroform technique with TRIzol reagent according to the manufacturer’s instructions. The quality and quantification were determined with spectrophotometric reading at 260, 280 and 230 nm using a NanoDrop 1000 Spectrophotometer (Thermo Fisher Scientific, Inc.). Retrotranscription using 5 μg of total RNA was achieved using the Transcriptor First Strand cDNA Synthesis kit primed with Oligo dT, according to the manufacturer's protocol. cDNA was used to confirm E6, E7, ERα, ERβ, GPER and PRLR expression using the LightCycler® FastStart DNA Master PLUS SYBR Green I kit on a LightCycler 2.0 instrument, according to the manufacturer's protocol. To normalize qPCR reactions, RPL32 expression was employed for HeLa and SiHa cells and ACTB for HaCaT transduced cells; the primers used to amplify the sequences are in Table 1. Thermocycling conditions were the following: One denaturation step at 95 °C for 10 min, followed by an amplification program of 40 cycles consisting of 95 °C for 10 s, 60 °C for 10 s, and 72 °C for 12 s, finally a final extension step at 72 °C for 10 min. The relative expression was calculated using the 2^−ΔΔCq^ and 2^−ΔCq^ methods.

**Table 1:** Primers employed in qPCR

Gen	Forward	Reverse
E6 HPV 16	5′ CAGACATTTTATGCACCA AA 3'	5′ CTCCATGCATGATTACAGC 3'
E7 HPV 16	5′ TAGAGAAACCCAGCTGTAATCA 3'	5′ AGGATCAGCCATGGTAGATTAT 3'
E6 HPV 18	5′ AATACTATGGCCGCTTTGA 3'	5′ TTGCCTTAGGTCCATGCATACT 3'
E7 HPV 18	5′ CGCAGAGAAACACAAGTATAAT 3'	5′ GATCAGCCATTGTTGCTTA 3'
RPL32	5′ GCATTGACAACAGGGTTCGTA G 3'	5′ ATTTAAACAGAAAACGTGCACA 3'
ESR1	5′-CCGGCTCCGTAAATGCTACG-3′	5′-TCCAGCAGACCCCACTTCAC-3’
ESR2	5′-TCGGAAGTGTTACGAAGTGGGAATGG-3′	5′-GCACTTCTCTGTCTCCGCACAA-3′
GPER	5^′^ AGTCGGATGTGAGGTTCAG 3^′^	5^′^ TCTGTGTGAGGAGTGCAA G 3^′^
PRLR	5´-AGTGAACTTCTGATACATTTCCTGC-3´	5´-TTGCAGATGCCACATTTTCCT-3´
ACTB	5′-CATGTACGTTGCTATCCAGGC-3’	5′-CTCCTTAATGTCACGCACGAT-3’

### Western blotting

40 µg of total protein extract from all cell lines (pLVX and cell lines transduced with the oncogenes) were mixed with loading buffer and denatured at 95 °C for 5 min. Afterward, electrophoresis was performed on 10% SDS polyacrylamide gels for proteins to be resolved. Transference to PVDF membranes was carried out for 1.5 h followed by a 24-h blocking step. Membranes were incubated in a primary antibody solution, diluted 1:500 and they were kept overnight. After incubation with secondary antibody (1:5000) membranes were revealed by a chemiluminescence system. MicroChemi 6.0 was used to reveal membranes and GelQuant software was used to analyze optical densitometry.

### Fluorescence immunocytochemistry

20,000 cells of each pLVX and cells transduced with the oncogenes were placed on 8-cell slides for 48 h. Cells were washed with PBS (0.2% albumin) and fixed with 4% paraformaldehyde for 10 min at room temperature. The cells were permeabilized with PBS (0.2% tween 20) for 10 min at 37 °C. Subsequently, the blocking step was carried out with PBS (10% FBS, 1% BSA) for 1 h at 37 °C. Antibodies were added with the following dilutions: PRL-R 1:50, ER-α 1:25, and ERβ 1:25. The incubation with the primary antibody was performed overnight, and then with the secondary antibody (1:2500) for 2 h and lastly, the nucleus was stained with DAPI (1:20,000) for 10 min and protected from light. Slides were observed under an Axio Imager 2 fluorescence microscope (Carl Zeiss, Gôttingen, Germany), using filters with the following excitation ranges: Alexa Fluor excitation: 495 nm, emission: 519 nm; DAPI excitation: 351 nm; emission: 461 nm. This analysis was performed with 2 independent assays for each cell line and at least 5 different fields were taken for each sample.

### Statistical analysis

Statistical assessment was conducted using GraphPad Prism 6 (GraphPad Software, Inc., La Jolla, CA, USA) statistical software. Data obtained from three independent tests were analyzed using a Kruskal Wallis test followed by unpaired t-test. The data are presented as the means ± standard deviation and statistically significant differences were considered for p-values < 0.05.

## Results

### PRL and E2 modulate the mRNA expression of E6 and E7 oncogenes on HeLa and SiHa cell lines

To evaluate whether PRL and E2 modulate the mRNA expression of E6 and E7 oncogenes on HeLa and SiHa cell lines, the qRT-PCR assay was performed in cells with hormonal stimuli for 4 h; the results were analyzed by the methods 2^−Δcq^ and 2^−ΔΔcq^, using RPL32 as the reference gene.

**Fig. 1:**
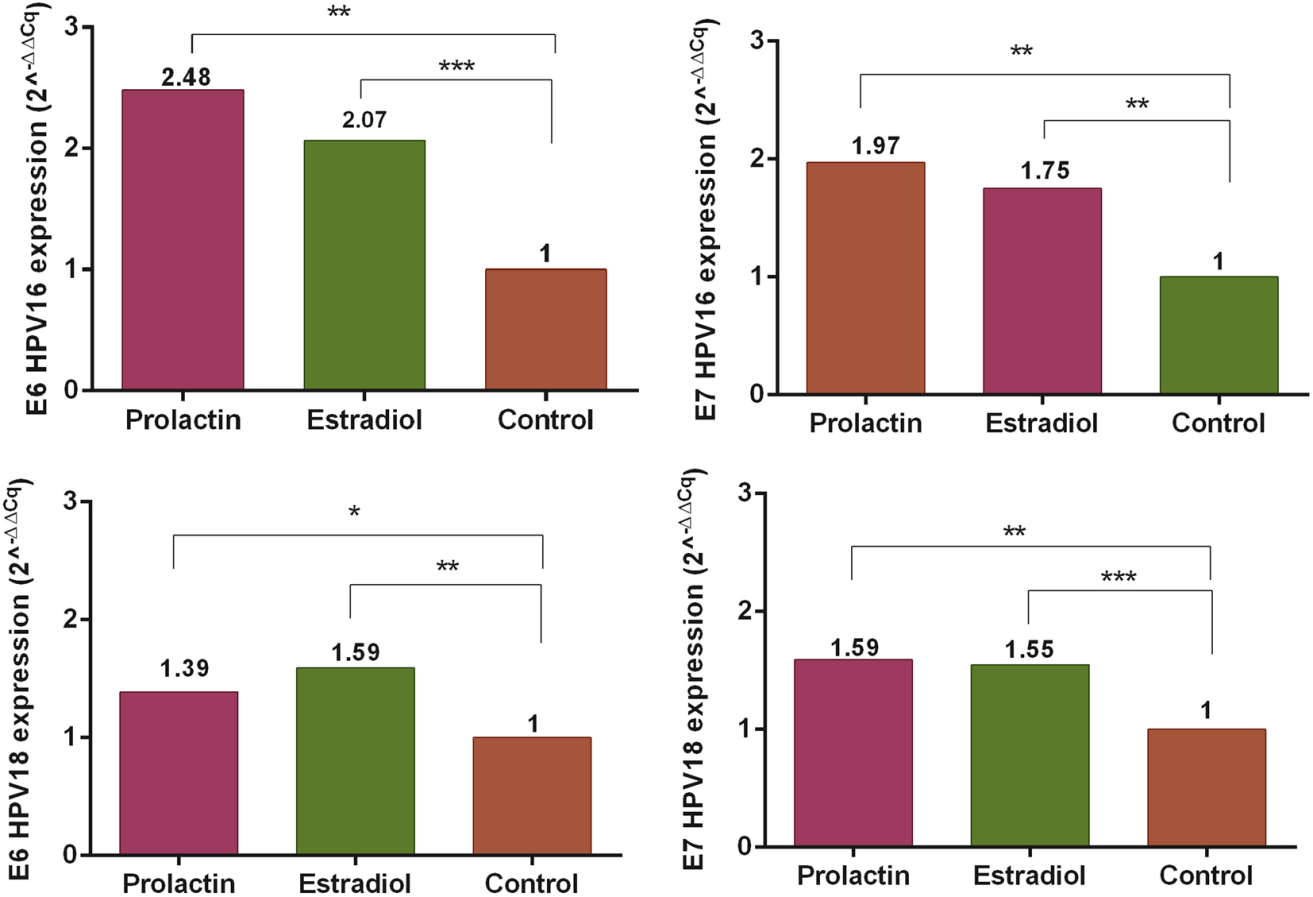
mRNA expression of E6 and E7 oncogenes from HPV-16 and -18 after prolactin and 17β-estradiol stimuli. HeLa and SiHa cells were stimulated with prolactin (200 ng/ml) and 17β-estradiol (10 nM) and relative mRNA expression were measured by quantitative PCR (qPCR) the graphics show the fold change calculated for the method 2^−ΔΔcq^ and the p-value calculated with 2^−Δcq^ method using RPL32 as the reference gene. (*p < 0.05; **p < 0.01; ***p < 0.001)

The relative expression was statistically analyzed through ANOVA-test. The graphics show the fold change calculated for the method 2^−ΔΔcq^ and the p-value calculated with 2^−Δcq^ method. In Fig. 1, it was observed that both E2 and PRL increased the expression of E6 and E7 oncogenes from HPV16 and HPV18 (p < 0.05).

### E6 and E7 oncogenes modulate the expression and localization of estrogen and PRL receptors

qPCR, western blot and, immunofluorescence assays were performed to evaluate if E6 and E7 oncogenes from HPV16 and HPV18 transduced in HaCaT cells can regulate the expression and localization of estrogen and PRL receptors.

The protein expression levels were measured through Western blot and immunofluorescence and the relative mRNA expression was calculated by qPCR using the 2^−ΔΔcq^ method. In addition, the expression of the nuclear receptors was measured in the immunofluorescence images. The p-value was calculated with ANOVA-test and the graphics show the expression in arbitrary optical density.

**Fig. 2:**
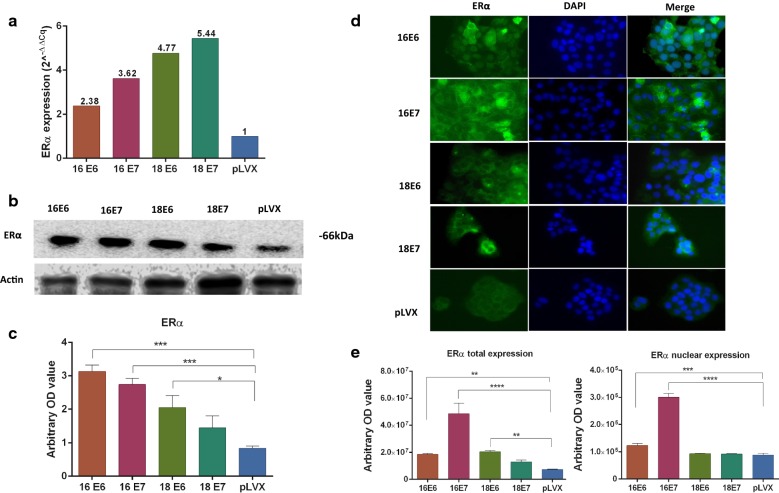
Expression and location of ERα in HaCaT cells transduced with E6 or E7 oncogenes from HPV-16 and 18. **a** Relative expression of ERα mRNA was measured by qPCR, the fold change was calculated by 2^−ΔΔcq^ method using ACTB as control. **b**, **c** ERα protein expression was determined by western blot, densitometric analysis was performed using β-actin as control. **d** Immunofluorescence was performed using a secondary antibody conjugated with Alexa Fluor 488 (Green) and DAPI stain (blue). Merged images are shown in magnification 40×. **e** The location of ERα was observed in cytoplasm and nuclei in immunofluorescence images (*p < 0.05; **p < 0.01; ***p < 0.001)

As can be seen in Fig. 2a, the E6 and E7 oncogenes from HPV16 and 18 enhance the mRNA expression of ERα in a range of 2.38–5.48 folds more than HaCaT cells transduced only with the lentiviral vector (pLVX). On the other hand, E6 and E7 oncogenes from HPV16, as well as the oncogene E6 from HPV 18, statistically significant increase the expression of 66 kDa isoform from ERα compared with pLVX (Fig. 2b, c). These results coincide with those observed in immunofluorescence and the localization of ERα change due to the presence of the different oncogenes. In pLVX cells and those with the E6 and E7 oncogenes of HPV18, ERα is located mainly in the cytoplasm of the cell. In contrast, the cells transduced with E6 and E7 oncogenes from HPV16 expressed a location of ERα predominantly in the nucleus (Fig. 2d, e).

**Fig. 3:**
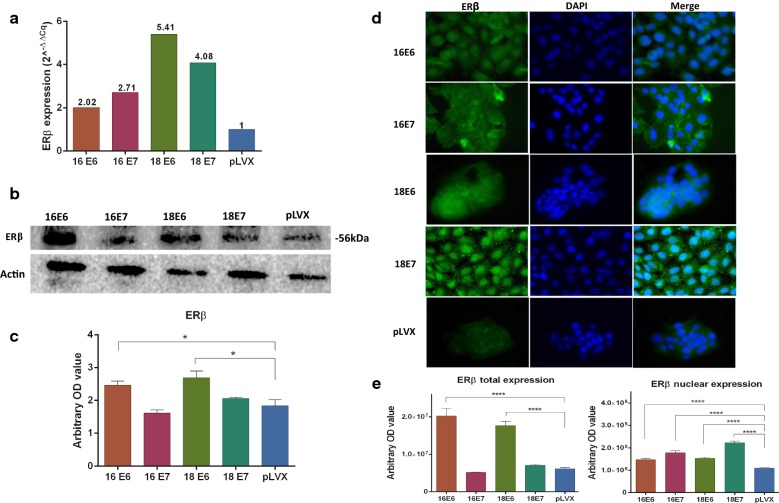
Expression and location of ERβ in HaCaT cells transduced with the E6 or E7 oncogenes from HPV-16 and 18. **a** Relative expression of ERβ mRNA was measured by qPCR, the fold change was calculated by 2^−ΔΔcq^ method using ACTB as control. **b**, **c** ERβ protein expression was determined with western blot, densitometric analysis was performed using β-actin as control. **d** immunofluorescence was done using a secondary antibody conjugated with Alexa Fluor 488 (Green) and DAPI stain (blue). Merged images are shown in magnification 40×. **e** The location of ERβ was observed in cytoplasm and nuclei in immunofluorescence images (*p < 0.05; **p < 0.01; ***p < 0.001)

The cells transduced with E6 and E7 oncogenes from both HPV increase the levels of ERβ mRNA in a range of 2.02–5.41 (Fig. 3a); the expression of a 56 kDa band of ERβ was observed in all HaCaT cell lines in the presence or absence of the oncogenes. However, the E6 oncogenes of HPV 16 and 18 induced greater and significant expression of the ERβ isoform, but in the presence of the oncogenes E7 the expression was similar to the pLVX cells (Fig. 3b, c). With respect to the localization of ERβ, in pLVX cells, it was mainly cytoplasmic, as well as for ERα. However, the E6 and E7 oncogenes of both HPVs induced greater expression of this receptor and a predominantly nuclear location although it is also observed in the cytoplasm (Fig. 3d, e).

**Fig. 4:**
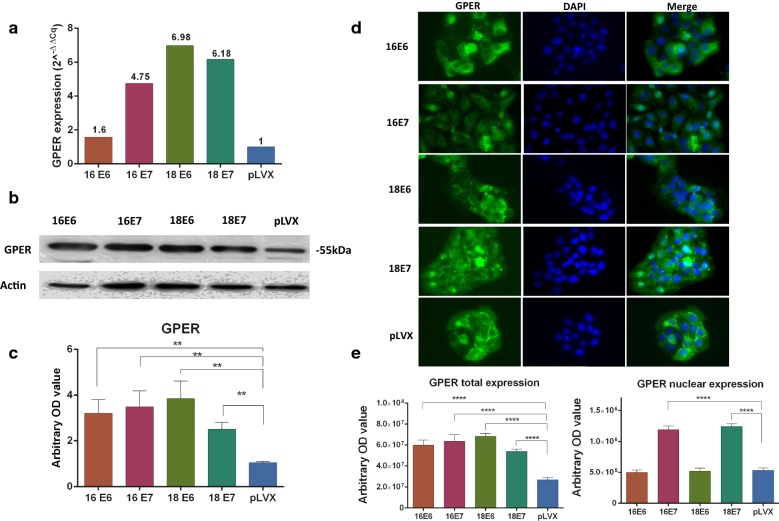
Expression and location of GPER in HaCaT cells transduced with the E6 or E7 oncogenes from HPV-16 and 18. **a** Relative expression of GPER mRNA was measured by qPCR, the fold change was calculated by 2^−ΔΔcq^ method using ACTB as control. **b**, **c** GPER protein expression was determined with western blot, densitometric analysis was performed using β-actin as control. **d** immunofluorescence was done using a secondary antibody conjugated with Alexa Fluor 488 (Green) and DAPI stain (blue), the images are shown in magnification 40×. **e** GPER location was observed in cytoplasm and nuclei in immunofluorescence images (*p < 0.05; **p < 0.01; ***p < 0.001)

GPER mRNA increased in cells transduced with E6 and E7 oncogenes from both HPV in a range of 1.6–6.98 folds (Fig. 4a), this finding correlated with the GPER protein levels detected in western blot analysis as a 55 kDa band in all cell lines (Fig. 4b, c) as well as with the expression in immunofluorescence (Fig. 4d, e). GPER location was found at both cytoplasm and nucleus, with the highest nuclear expression in the case of cells transduced with E7 oncogene of both HPV in comparison with pLVX cells; meanwhile, E6 oncogenes promoted a predominantly cytoplasmic expression (Fig. 4d, e).

**Fig. 5:**
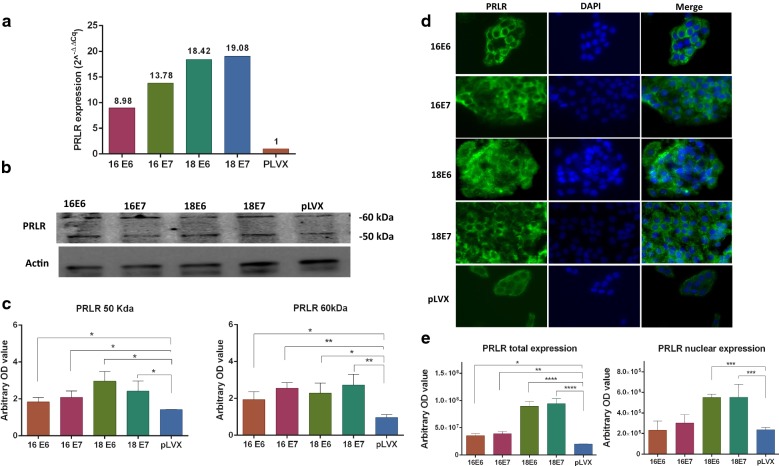
Expression and location of PRLR in HaCaT cells transduced with the E6 or E7 oncogenes from HPV-16 and 18. **a** Relative expression of PRLR mRNA was measured by qPCR, the fold change was calculated by 2^−ΔΔcq^ method using ACTB as control. **b**, **c** PRLR protein expression was determined by western blot, densitometric analysis was performed using β-actin as control. **d** PRLR immunofluorescence was done using a secondary antibody conjugated with Alexa Fluor 488 (Green) and DAPI stain (blue), the images are shown in magnification 40×. **e** PRLR location was observed in cytoplasm and nuclei in immunofluorescence images (*p < 0.05; **p < 0.01; ***p < 0.001)

The mRNA PRLR expression was evaluated with primers that detect all isoforms, and we found that E6 and E7 oncogenes from both, HPV16 and 18, increased the levels in 8.98–19.08 folds more than the control (Fig. 5a). Previously, we reported that HaCaT cells express the low molecular weight isoforms of PRLR (50 and 60 kDa) in a very mild way [13]. However, the E6 and E7 oncogenes of both HPV induced a greater expression that was evidenced by western blot (Fig. 5b, c) and by immunofluorescence (Fig. 5d, e). This receptor was localized in the cell membrane and cytoplasm of control and oncogene transduced cells, surprisingly, the expression of E6 and E7 oncogenes from only HPV18 induced the nuclear localization of PRLR (Fig. 5d, f).

## Discussion

HPV is considered the main causal agent for the development of CC and despite this, it is known that infection with this virus is insufficient for the development of this disease. Therefore, other factors are required, among them the hormonal ones that cooperate for their permanence and the beginning of the malignant transformation [4].

While most murine models confirm that cervical carcinomas are addicted to estrogens, and especially to their ERα, few human studies show that their expression declines in cancer [4, 5, 7, 18, 19]. However, we have recently observed that both, ERα and ERβ, nuclear receptors are overexpressed in CC and its precursor lesions, and their expression increases gradually as lesions progress to CC [8]. Likewise, the GPER expression is also increased in CC in comparison with premalignant lesions; the location of this receptor was predominantly in the cytoplasm, although it was also evidenced in the nucleus of the epithelial cells [14]. Other reports had shown that the GPER is located in the membrane and cytoplasm of CC tissue samples; however, its location in the nucleus was not observed [10]. In the case of breast tumors, an expression of GPER in the cytoplasm was associated with low-stage histological subtypes and the nuclear location was associated with poorly differentiated carcinomas, which leads to conclude that nuclear location may have unfavorable tumor properties [20]. On the other hand, the expression of PRL at a systemic level in patients with CC also reveals contradictory results. We recently identified a 60 kDa PRL secreted by cells derived from CC that is bioactive [12]. In addition, we observed high expression of PRLR in CC as opposed to premalignant lesions [11].

Although there are not many studies that demonstrate the participation of estrogen and prolactin in cervical carcinogenesis, these have also been related to the development of mammary tumors and their receptors are an important therapeutic target in breast cancer. It has been found that, as in CC, there is an autocrine secretion of prolactin in the tumor cells, and the activation of PRLR triggers the signaling pathway of Src-ERK-AKT and phosphorylate the ERα inducing the activation and recruitment of the receptor to the ERE [20, 24–26]. On the other hand, it is known that the alone expression of ERα or PRLR is not related to affect the progression of the tumor, whereas the co-expression of both receptors is associated to the induction of invasion and a reduced response to estrogen antagonists. In CC, PRLR and ERα are also co-expressed, which indicates that there may be an interaction between both receptors [13, 27–29].

E2 and 60 kDa PRL have no direct effect on cell survival as assessed by the processes of proliferation and apoptosis; however, both hormones have an effect on the cellular metabolism of cell lines derived from CC positive for HPV infection [8].

Although the estrogen and prolactin receptors are co-expressed in CC, a persistent HPV infection is present in almost all cases of this type of cancer [8]. For this reason, it is interesting to evaluate the interaction between these hormones with HPV and its involvement in modulating the expression of their receptors, as well as evaluating how these hormones may be cooperating with HPV to modify its regulation and favor the persistence of infection and tumor progression.

E2 increases the transcription of important oncogenes of the HPV, resulting in the degradation of p53 and cell cycle alterations [17]; however, the role that PRL could have on this process is unknown. Here we show that both, E2 and PRL, have the capacity to increase the expression of E6 and E7 oncogenes in SiHa and HeLa cells that have an infection by HPV 16 and 18, respectively.

It is known that the genome of HPV 16 has two sites of EREs [17]; however, its functional significance remains to be elucidated.

Viral oncogenes directly deregulate ER coactivators by altering gene expression [21]. We demonstrated that E6 and E7 oncogenes from HPV 16 and 18 are able to increase the expression and modify the location of hormone receptors. In the case of estrogen receptors, the oncogenes locate them in the nucleus and cytoplasm, and they increase the expression and cytoplasmic localization of the PRLR.

These changes in the location of the receptors lead us to suppose that signaling pathways related to metabolic and hormonal regulation could be activated, as well as HPV oncogenes activation. However, further analyses are necessary to find this out.

The physiological relationship between estrogen and prolactin has been well demonstrated [15, 16]; however, the impact of this relationship on cancer has been little explored. Numerous studies have accepted the synergism between estrogen and PRL in the promotion of breast cancer and the ability of PRL to activate ERα in the absence of its natural ligand [14, 15]. This leads us to suppose that one of the ways that PRL could use to activate the E6/E7 oncogenes of HPV would be through the activation of ERα. It is known that E2 through ERα is essential for the transition to cancer observed in a model of mice infected with the E6 and E7 oncogenes of HPV16 [7].

## Conclusion

Taken together, our results suggest that cooperation between E2, PRL, and HPV is necessary to contribute to the development of CC. Therefore, the interaction of these factors that are co-expressed in advanced cervical lesions should be more deeply researched in order to have a better understanding of this hormone-HPV oncogenic regulation.

## Data Availability

Not applicable.

## Abbreviations

CC: cervical cancer
E2: 17β-estradiol
PRL: prolactin
PRLR: prolactin receptor
ERα: estrogen receptor alpha
ERβ: estrogen receptor beta
GPER: g protein-coupled estrogen receptor
